# Extraperitoneal tissue retraction technique: An effective assistant of extraperitoneal pure single-port robotic-assisted radical prostatectomy with the da Vinci Si surgical system

**DOI:** 10.3389/fsurg.2022.941104

**Published:** 2022-10-25

**Authors:** Guanqun Ju, Zhijun Wang, Jiazi Shi, Weidong Xu, Zongqin Zhang, Lei Yin, Dongliang Xu, Shancheng Ren

**Affiliations:** ^1^ Department of Urology, Second Affiliated Hospital of Naval Medical University, Shanghai, China; ^2^ Urology Centre, Shuguang Hospital Affiliated to Shanghai University of Traditional Chinese Medicine, Shanghai, China

**Keywords:** extraperitoneal tissue retraction technique, single-port surgery, robotic-assisted radical prostatectomy, extraperitoneal pathway, minimally invasive surgery

## Abstract

**Objective:**

The limitations of tissue retraction and the amount of surgical working space have a great impact on extraperitoneal single-port robotic-assisted radical prostatectomy (sp-RARP) with the multiport robotic surgical system. We used an extraperitoneal tissue retraction technique to achieve tissue exposure and working space expansion. This study evaluated the safety, feasibility, and efficacy of the extraperitoneal tissue retraction technique in extraperitoneal pure sp-RARP with the da Vinci Si surgical system.

**Methods:**

Data from 42 patients were analyzed retrospectively from December 2018 to February 2020. The extraperitoneal tissue retraction technique was not used in 20 patients (group I) and was used in 22 patients (group II). Preoperative, intraoperative, and postoperative data were collected. The oncological and functional data during late follow-up were recorded.

**Results:**

All patients successfully underwent extraperitoneal pure sp-RARP. No patients required conversion to a multiport surgery or placement of additional assistant ports. The two groups were similar regarding baseline features. The median operation time in group I was significantly longer than that in group II (*P* < 0.001). The estimated blood loss volume in group I was significantly higher than that in group II (*P* < 0.001). There were no serious complications in either group. There were four cases of peritoneal tears in group I and none in group II (*P* = 0.043). The surgical margin and lymph nodes were negative in both groups. The oncological and functional outcomes were similar between the two groups 6 months after the procedure.

**Conclusions:**

The extraperitoneal tissue retraction technique is safe and feasible. The technique promotes tissue exposure and expands the surgical working space, which is important for achieving extraperitoneal pure sp-RARP with the da Vinci Si surgical system, especially for beginners. The short-term oncological and functional outcomes were within acceptable ranges. The long-term effects of this technique need further evaluation.

## Introduction

Robot-assisted radical prostatectomy (RARP) is the standard treatment for localized prostate cancer (PCa) (1, 2). Currently, we are in a technology-driven era aimed at balancing the maximization of oncological results with the minimization of surgery-related impacts on patient quality of life (3, 4). Single-port RARP (sp-RARP) is considered the direction for future development (5, 6). The use of single-port surgery can not only reduce trauma and speed up postoperative recovery but also increase patient satisfaction in terms of cosmetics and reduce their psychological trauma (4, 7, 8).

The new da Vinci SP surgical system has shown great potential for sp-RARP (9, 10). However, the SP platform is not approved for use in China. Most medical centers still use the da Vinci Si/Xi system for RP. These platforms are not specifically designed for single-port surgery, and many restrictions are associated with single-port surgery (11–13). In a previous study, we successfully completed a series of extraperitoneal pure sp-RARP procedures without adding an auxiliary port using the da Vinci Si system and showed its advantages in terms of cosmetics, pain, and postoperative recovery time, which was rapid (14, 15). Due to the lack of a fourth robotic arm, we found that tissue exposure and working space constraints posed great challenges to the use of extraperitoneal pure sp-RARP. The need for a means of tissue retraction that can overcome exposure and challenges with extraperitoneal space was evident. By learning from the experience of White et al. (11) and through refinements of the technique, we adopted an extraperitoneal tissue retraction technique to help operators achieve extraperitoneal pure sp-RARP.

The purpose of this study was to verify the safety and feasibility and describe the details of the extraperitoneal tissue retraction technique. We hope to better define the precise role of the extraperitoneal tissue retraction technique and encourage more doctors to explore the use of extraperitoneal pure sp-RARP.

## Materials and methods

### Patients and data collection

The data of 42 **consecutive** patients with localized PCa who underwent extraperitoneal pure sp-RARP from December 2018 to February 2020 were analyzed retrospectively. Twenty patients were not treated with the extraperitoneal tissue suspension technique (group I), and 22 patients were treated with the extraperitoneal tissue suspension technique (group II). The exclusion criteria included a previous infraumbilical midline incision, body mass index (BMI) of >40 kg/m^2^, preoperative prostate-specific antigen (PSA) levels of >20 ng/ml, biopsy Gleason score of >7, prior prostate treatment, or preoperative evidence of extraprostatic disease. Preoperative assessment, staging, and risk stratification were performed using physical examination, prostate biopsy results, PSA levels, and imaging examination. After a comprehensive discussion, informed consent was obtained.

The baseline characteristics of the patients, operation time (from skin incision to skin closure), estimated blood loss (EBL), peritoneal tear, intraoperative complications, and duration of hospital stay were collected. Complications were assessed intraoperatively or postoperatively using the Clavien–Dindo classification system and were classified as major (grade ≥III) or minor (grade ≤II) (16). Pathology data, including the final pathological stage, positive surgical margins (PSM), and lymph node invasion, were recorded. All patients were followed up regularly to monitor the state of urinary continence and biochemical recurrence. Urinary continence was evaluated by the number of urine pads used per day (17). No use or the use of no more than one pad/day was considered to reflect good urinary continence. All the patients underwent sp-RARP by two surgeons who were experienced in multiport robotic surgery but did not go beyond their learning curve with sp-RARP. The patients signed a written agreement to participate. The patients undergoing single-port surgery were advised to receive additional assistance ports as needed during the operation.

### Surgical procedure

**Figure 1:**
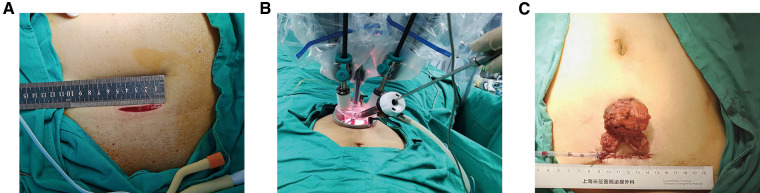
Illustration of extraperitoneal pure sp-RARP. (**A**) A 4–5 cm transverse abdominal incision approximately 5 cm above the pubic symphysis. (**B**) Intraoperative installation showing a scope holder arm and the two primary robotic arms. (**C**) Wound closure with drainage placed in the single-port incision. sp-RARP, single-port robotic-assisted radical prostatectomy.

All patients underwent surgery with the da Vinci Si surgical system (Intuitive Surgical, Sunnyvale, CA, United States). A 4–5 cm transverse incision was made approximately 5 cm above the pubic symphysis (Figure 1A). Then, an 8-cm quadri-channel laparoscopic port (Lagis Inc., Taichung, China) was placed. The scope holder arm and two primary robotic arms were used (Figure 1B). The patients were placed in a low lithotomy position with no steep Trendelenburg position. Prophylactic single-dose intravenous antibiotics (e.g., cephalosporins) and subcutaneous prophylactic heparin (2,000 IU) were administered prior to surgery. Patients with PSA levels of >10 ng/ml underwent pelvic lymph node dissection. The main surgical steps of the operation were described in our previous report (14). A drainage tube was routinely placed (Figure 1C).

### Key procedure of the extraperitoneal tissue retraction technique

**Figure 2:**
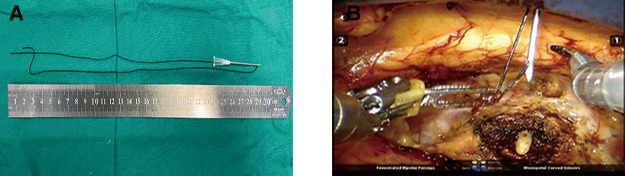
Homemade abdominal puncture device and its application points. (**A**) The abdominal puncture needle was made by inserting retraction sutures into the needle of a 20 ml syringe. (**B**) The needle was inserted vertically into the abdominal wall near the midline of the pubic symphysis, and the retraction sutures were pulled out by a robotic arm.

The marionette technique was carried out by inserting retraction sutures into the needle of a 20 ml syringe (Figure 2A). When the tissue needed to be retracted, the needle was inserted vertically into the abdominal wall close to the midline of the pubic symphysis, and the retraction sutures were pulled out and fixed onto the tissue through a hem-o-lock (Figure 2B). The retraction sutures were fixed outside the abdominal wall with a vascular clamp. The position of the sutures was adjusted to retract the desired tissues according to the operation procedure.

**Figure 3:**
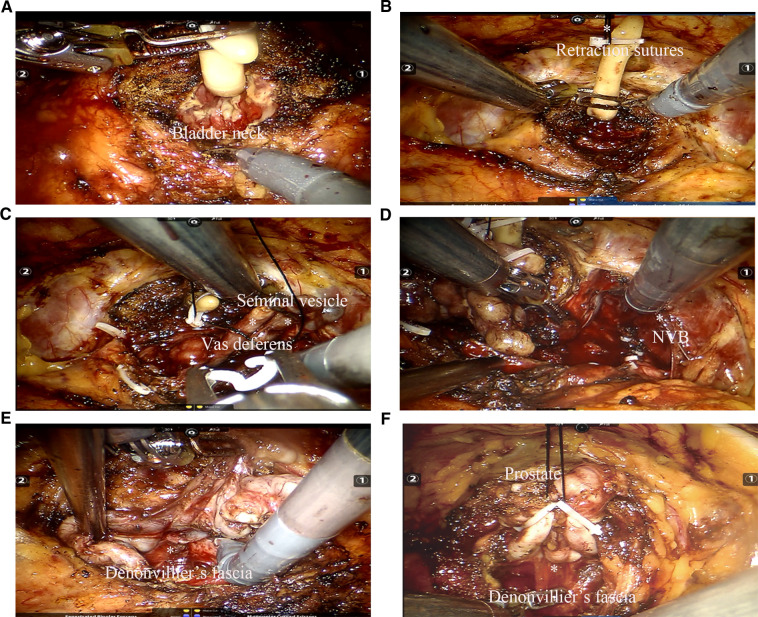
Key procedure of the extraperitoneal tissue retraction technique. (**A**) Because the assistance of the fourth arm in extraperitoneal pure sp-RARP was absent, one robotic arm needed to act as a retractor to stretch the catheter. (**B**) The retraction sutures and catheter were fixed together during dissection of the bladder neck. (**C**) The retraction sutures and prostate needed to be fixed together, which facilitates the dissection of the vas deferens and seminal vesicles. (**D**) During dissection of the NVB, the prostate, seminal vesicles, and vas deferens were suspended. (**E**) Use of robotic arms to retract the ampullae and their attached seminal vesicles anterior to help identify the anterior rectal wall in extraperitoneal pure sp-RARP. (**F**) The prostate, seminal vesicles, and vas deferens were retracted to expose the visual field and visualize Denonvilliers' fascia. sp-RARP: single-port robotic-assisted radical prostatectomy; NVB: neurovascular bundles.

An extraperitoneal tissue retraction technique might be required at some critical stages of extraperitoneal pure sp-RARP. First, during bladder neck dissection, retractor pressure was applied to the bladder and catheter, which facilitated visualization of the vesicoprostatic junction. Without the assistance of the fourth arm in the extraperitoneal pure sp-RARP, one robotic arm needed to act as a retractor, which might have increased the risk of detrusor fiber damage and bleeding (Figure 3A). We fixed the retraction sutures and the catheter together to ensure a certain suspension tension (Figure 3B), which was used to replace the role of the fourth arm in conventional RARP. Second, the surgical field needed not to be disturbed when separating the vas deferens and seminal vesicles. A clear surgical field could be maintained with the use of conventional RARP with the help of the assistant port and the fourth arm, which were absent in sp-RARP. We needed the help of an extraperitoneal tissue retraction technique to expose the space. When one side of the seminal vesicle and vas deferens were dissected, the retraction sutures, dissected seminal vesicle, and vas deferens were suspended together. This approach was beneficial to the dissection of the vas deferens and seminal vesicles on the other side (Figure 3C). Third, in the process of the neurovascular bundle (NVB) sparing, because of the need for multiple operations and fine movements, we required greater exposure to the surgical visual field. The prostate, seminal vesicles, and vas deferens needed to be retracted using the extraperitoneal tissue retraction technique (Figure 3D). Finally, separating the ampullae from the seminal vesicles could challenge the visualization of the posterior Denonvilliers' fascia, particularly in patients with large seminal vesicles. Robotic arms were used to retract the ampullae and their attached seminal vesicles anteriorly to help identify the anterior rectal wall in epR-spRP (Figure 3E). Incising Denonvilliers' fascia with one remaining robotic arm increases the risk of rectal injury by entering the wrong layer, and especially increases the risk of tissue adhesion. The sutures were used to retract the seminal vesicle and vas deferens anteriorly, which promoted Denonvilliers' fascia exposure and reduced rectal injury (Figure 3F). The key procedures of the extraperitoneal tissue retraction technique were shown in the video as Supplementary material.

### Statistical analyses

Normally distributed data were analyzed with the one-sample Kolmogorov–Smirnov test. Univariate analysis was performed using parametric (Student's *t*-test) and nonparametric (Mann–Whitney *U* test) tests for continuous variables, and the chi-square test (or Fisher exact test) was used for categorical variables, as appropriate. Statistical significance was set at *P* < 0.05.

## Results

### Baseline characteristics

There was no difference in age, BMI, preoperative PSA levels, or clinical stage between the two groups. We routinely performed prostate magnetic resonance imaging and whole-body bone scans before RP to rule out the occurrence of local metastasis and bone metastasis. Preoperative prostate magnetic resonance imaging and whole-body bone scans showed no metastasis in any patient. According to the D’Amico risk classification for PCa (18), there were eight low-risk patients in group I and seven low-risk patients in group II, and all the others were medium-risk patients. In group I, four patients (20.0%) had a history of abdominal surgery. In group II, five patients (22.7%) had a history of abdominal surgery. The perioperative baseline characteristics of the patients are shown in Table 1.

**Table 1:** Preoperative patient characteristics.

Parameter	Group I (*n* = 20)	Group II (*n* = 22)	*P*-value
Age (y), mean ± SD	67.3 ± 4.8	66.5 ± 4.5	0.559
BMI (kg/m^2^), mean ± SD	25.1 ± 2	25.3 ± 1.7	0.704
Prior abdominal surgery, *n* (%)	4 (20.0)	5 (22.7)	>0.999
PSA (ng/ml), median (IQR)	9.7 (8.7–11.7)	9.6 (8.9–11.4)	0.820
cT stage, *n* (%)			>0.999
cT1c	6 (30.0)	6 (27.3)	
cT2a	10 (50.0)	12 (54.5)	
cT2b	4 (20.0)	4 (18.2)	
Biopsy ISUP grade, *n* (%)			0.591
Grade group1	4 (20.0)	4 (18.2)	
Grade group2	6 (30.0)	4 (18.2)	
Grade group3	10 (50.0)	14 (63.6)	
D’Amico risk classification, *n* (%)			0.749
Low-risk	8 (40.0)	7 (31.8)	
Medium-risk	12 (60.0)	15 (68.2)	

IQR, interquartile range; BMI, body mass index; PSA, prostate-specific antigen; ISUP, International Society of Urological Pathology.

### Surgical outcomes

All patients successfully underwent extraperitoneal pure sp-RARP. No patient required additional port placement. No patient's treatment was changed to multiport RARP or open surgery. Lymph node dissection was performed in 12 patients in group I and 15 patients in group II. The median operation time in group I was significantly longer than that in group II (175.0 vs. 131.5 min; *P* < 0.001). The EBL in group I was also significantly higher than that in group II (163.4 vs. 117.6 ml, *P* < 0.001). No patient required transfusion. There was no difference in the duration of hospital stay between the two groups (*P* = 0.519). The incidence of complications in the two groups was similar, and there were no complications worse than Clavien–Dindo grade II. NVB sparing was not significantly different between the groups. There were four cases of peritoneal tears in group I and none in group II (*P* = 0.043). The surgical margin and lymph nodes were negative in both groups. The surgical results and postoperative pathological stages are shown in Table 2.

**Table 2:** Comparisons of intraoperative and postoperative data, complications between not using extraperitoneal tissue retraction technique (group I) and using extraperitoneal tissue retraction technique (group II).

Parameter	Group I (*n* = 20)	Group II (*n* = 22)	*P*-value
Operative time (min), median (IQR)	175.0 (168–183.8)	131.5 (120.8–137.3)	<0.001
EBL (ml), mean ± SD	163.4 ± 17.7	117.6 ± 16.6	<0.001
Bladder catheterization (d), median (IQR)	7	7	** **
PSM, *n* (%)	0	0	** **
Hospital stay (d), median (IQR)	5 (4.3–6)	5 (4–6)	0.519
Transfusion rate, *n* (%)	0	0	
peritoneal tear, *n* (%)	4 (20.0)	0	0.043
Lymphadenectomy, *n* (%)	12 (60.0)	15 (68.2)	0.749
Lymph node invasion, *n* (%)	0	0	
NVB-sparing procedure, *n* (%)			0.841
Unilateral	2 (10.0)	2 (9.1)	
Bilateral	16 (80.0)	19 (86.4)	** **
None	2 (10.0)	1 (4.5)	
Complications, *n* (%)			>0.999
Clavien I–II	6 (30.0)	7 (31.8)	
Clavien III–V	0	0	** **
ISUP grade after RP, *n* (%)			0.986
Grade group1	2 (10.0)	3 (13.6)	** **
Grade group2	4 (15.0)	4 (18.2)	** **
Grade group3	12 (60.0)	13 (59.1)	** **
Grade group4	2 (10.0)	2 (9.1)	
pT stage after RP, *n* (%)			>0.999
T2	18 (90.0)	19 (86.4)	
T3a	2 (10.0)	2 (9.1)	** **
T3b	0	1 (4.5)	
6-mo PSA<0.1 ng/ml, *n* (%)	18 (90.0)	20 (90.9)	>0.999
6-mo continence (0–1 pad/day), *n* (%)	16 (80.0)	19 (86.4)	0.691

QR, interquartile range; EBL, estimated blood loss; PSM, positive surgical margin; NVB, neurovascular bundle; RP, radical prostatectomy; PSA, prostate-specific antigen; ISUP, International Society of Urological Pathology.

### Six-month oncological and functional outcomes

All patients had enough follow-up for 6-month postoperative PSA levels and continence data. The rate of PSA levels of <0.1 ng/ml was over 90% in both groups (*P* > 0.999). The rate of patients who used 0–1 pads/day was not different between the two groups (*P* = 0.691). The oncological and functional results are shown in Table 2.

## Discussion

The advantages of robotic surgical systems include reduced instrument crossover, excellent ergonomic value, restored instrument triangulation, and improved effectiveness of single-port surgery (19, 20). The extraperitoneal approach RARP avoids entering the peritoneal cavity has minimal influence on intestinal function, and does not require a steep Trendelenburg position, which is more beneficial for postoperative recovery (21, 22). However, there are significant limitations to using a multiport surgical platform for single-port surgery, especially during instrument collisions and intraoperative sutures (20). Furthermore, the extraperitoneal working space is narrower and more limited (23). Expanding the working space and exposing the tissue are very important for the use of extraperitoneal pure sp-RARP with the da Vinci Si surgical system. In the absence of the new SP platform, we believe that the extraperitoneal tissue retraction technique is an effective method for solving the problem of limited surgical working space and tissue retraction in extraperitoneal pure sp-RARP, especially for beginners.

At present, a straight needle is used in some tissue retraction methods (10, 11). Nevertheless, the straight needle is relatively long and needs to pass through the abdominal wall twice, which may lead to an increase in the risk of injury in the narrow extraperitoneal space. Steinberg et al. (24) used a magnetic retractor to assist in tissue retraction in robotic prostatectomy with the new SP system. We used a 20 ml syringe needle as a handy tool to assist in tissue retraction. The length of the needle is very suitable for the depth of the extraperitoneal space. The needle does not need to be removed from the abdominal wall twice, which reduces the risk of inferior epigastric vessel injury. The surgeon can adjust the position of the suture according to the operation process to make the surgery more autonomous. However, our tissue retraction equipment was easy to manufacture and conferred no additional cost.

The operation time and bleeding volume are important indicators of the safety and feasibility of a technique. Wilson et al. reported that the operation time and EBL associated with extraperitoneal radical prostatectomy *via* the SP platform were 198.0 min and 179 ml, respectively (25). A high-volume surgical center showed that the average operation time and average EBL of conventional extraperitoneal robotic RP were 146 min and 100 ml, respectively (26). The operation time (175.0 min) and EBL (163.4 ml) exceeded our expectations in patients who did not receive the extraperitoneal tissue retraction technique in our study. Due to the lack of the fourth arm and additional assistant ports, tissue retraction and space exposure of the narrow pelvis are strictly limited. Additionally, the collision of instruments occurs in the upright environment of the robotic arm. The bedside assistant cannot be used to focus on suction/irrigation and clip application. The unstable visual field of the operation may be an important factor leading to an increase in operation time and bleeding volume. The extraperitoneal tissue retraction technique benefits tissue exposure, increases the surgical space, and maintains a stable surgical view. The results showed that the operation time and EBL decreased significantly after the extraperitoneal tissue retraction technique.

Injury to inferior epigastric vessels caused by abdominal puncture is also a concern (27). There was no inferior epigastric vessel injury or bleeding in our patients that were subjected to the extraperitoneal tissue retraction technique. Our experience was that the puncture point was as close to the midline of the pubic symphysis as possible, and the needle was inserted vertically. In our study, no major complications were observed in either group. Although no significant reduction in NVB sparing was observed in patients that did not undergo the extraperitoneal tissue retraction technique, more time was required to eliminate interference and perform a detailed dissection. This finding might be related to the reduction in visual field interference that occurs with the extraperitoneal tissue retraction technique.

The PSM rate is our key concern and is directly related to the prognosis of PCa patients (28, 29). PSM was present in up to 17% of patients when experienced surgeons performed RP (30). In this study, there were no patients with PSM in either group. The rate of patients with PSA levels of <0.1 ng/ml in both groups was over 90% during the short follow-up period. More than 80% of the patients recovered continence within 6 months. The low PSM rate, the short-term oncological outcomes, and the recovery of urinary continence may be related to the proportion of low/medium-risk patients and the insufficient sample size in our study.

The limitations of this study should be mentioned. Overall, this was a retrospective study, and the sample size was small. The patients who underwent extraperitoneal pure sp-RARP with the da Vinci Si surgical system were highly screened individuals and adopted a relatively conservative treatment approach. Therefore, these results were preliminary, and the risk of selection bias was inevitable. During the study, we determined the trends in the clinical benefits provided by the extraperitoneal tissue retraction technique. Thus, only a small number of patients were not treated with the extraperitoneal tissue retraction technique, which may have caused bias in the results and weakened the conclusions regarding oncological and functional outcomes. However, this was considered the best course of action for the patients. This approach is still being investigated, and we will consider these limitations in further studies in a larger cohort of patients. We believe that this technique could be used in other single-port operations, such as during retroperitoneal single-port kidney and gynecological surgery. We encourage more doctors to explore the limitations of the technology and assess its potential benefits.

## Conclusions

The extraperitoneal tissue retraction technique facilitates tissue exposure and expands the operation space in extraperitoneal pure sp-RARP with the da Vinci Si surgical system. It is safe, feasible, and effective, especially for beginners, to complete extraperitoneal pure sp-RARP. The short-term oncological and functional were promising but will require longer-term follow-up. Although we lack a new SP platform, we have demonstrated our commitment to maximizing the clinical benefit for patients. Randomized trials with adequate sample sizes and postoperative follow-up periods are necessary.

## Data Availability

The raw data supporting the conclusions of this article will be made available by the authors, without undue reservation.
